# WELD-DETR: A Real-Time Welding Defect Detection Framework with Multi-Scale Feature Fusion and Multi-Kernel Perception Optimization

**DOI:** 10.3390/s25227024

**Published:** 2025-11-17

**Authors:** Yu Liang, Bojian Yu, Mengyu Ding, Wenchen Hu, Yuchang Jin, Yuefeng Yuan

**Affiliations:** School of Marine Engineering Equipment, Zhejiang Ocean University, Zhoushan 316022, China224473449@st.usst.edu.cn (B.Y.);

**Keywords:** X-ray images analysis, automated defect recognition, welding defects, deep learning

## Abstract

Welding technology plays a crucial role in manufacturing, aerospace, construction, and military industries, where the quality of welds directly impacts the safety and reliability of overall structures. Therefore, developing a real-time welding defect detection framework based on deep learning is of paramount importance. However, existing detection methods suffer from limitations such as insufficient real-time performance, high miss rates for small defects, and poor adaptability to complex working conditions. To address these challenges, this paper proposes a welding defect detection framework named WELD-DETR, which incorporates multi-scale feature fusion and multi-kernel perception collaborative optimization. First, we introduce a novel hierarchical feature pyramid (HFPS) structure that effectively combines low-level high-resolution features with high-level semantic features, significantly improving the detection rate of micron-level cracks and pores. Secondly, we innovatively design a multi-kernel perception wavelet convolution (MPWC) module to enhance the model’s ability to respond to edge features and fine textures at various scales. Finally, to further boost the model’s generalization capability, we construct an industrial-grade welding dataset encompassing five common defect types and propose a cross-condition training strategy based on transfer learning. Experimental results show that WELD-DETR achieves an mAP@0.5–0.95 of 98.2%, a precision of 96.8%, and an inference speed of 58 FPS on an RTX 2060 GPU. Moreover, it exhibits superior detection accuracy and real-time performance in complex industrial scenarios such as high noise and strong reflections, outperforming existing state-of-the-art methods in accuracy. These results underscore WELD-DETR’s potential to support intelligent welding quality assurance and process optimization in real-world applications.

## 1. Introduction

Welding technology is a cornerstone in modern manufacturing, construction, and aerospace industries [1]. This study focuses on the detection of defects in arc-welded joints of low-carbon steel structures, which are extensively used in heavy manufacturing and engineering applications. The challenges in identifying micron-scale defects in such components using X-ray imaging constitute the primary motivation for this work. By heating metal materials to high temperatures, welding fuses different components together to form strong joints [2]. A wide range of welding methods exist, including arc welding, gas welding, laser welding, and spot welding, each with unique advantages and application scenarios [3,4]. This work specifically addresses arc welding due to its prevalence in industrial settings where internal defect detection through X-ray radiography plays a crucial role in quality control. However, in real-world welding operations, particularly in the gas metal arc welding (GMAW) of low-carbon steel structures investigated in this study, the process is often affected by both the inherent properties of materials and external environmental factors, leading to common quality issues such as porosity, cracks, and lack of fusion [5]. These defects are among the most critical for this specific welding method and material combination. These defects not only compromise the appearance of welded products but also directly impact the mechanical performance of welded joints. This is particularly critical in fields such as aerospace and nuclear power, where safety requirements are extremely stringent [6]. Welding defects can weaken the overall structural strength and may result in fatigue failure or catastrophic malfunction during operation. Therefore, timely and accurate detection of welding defects has become an essential means of ensuring product quality and structural safety [7].

To guarantee weld quality, traditional inspection methods such as visual testing, ultrasonic testing and magnetic particle testing are still widely used [8]. While these approaches can effectively detect certain surface defects, they exhibit considerable limitations when faced with complex weld geometries or hidden internal flaws. For multilayer welds or deep subsurface defects in particular, conventional methods often fail to provide comprehensive and precise identification and localization. This shortcoming introduces potential quality risks and, in severe cases, may even compromise the safety of entire engineering structures.

As a result, X-ray inspection has become an important technique in weld defect detection, owing to its strong penetration capability and high-resolution imaging. By utilizing X-ray imaging, internal flaws such as porosity, cracks, and inclusions can be revealed without damaging the integrity of the product, thereby ensuring structural safety [9]. This non-destructive testing method provides clear images of internal weld quality, serving as a crucial basis for evaluating joint reliability [10]. Nevertheless, despite its advantages, X-ray inspection still has inherent limitations. Under conditions such as complex welded components or large-scale industrial production environments, conventional X-ray methods struggle to meet the demands for both efficiency and precision. Moreover, due to the complex formation mechanisms of weld defects and the reliance on operator subjectivity, reliable interpretation of radiographs remains challenging [11].

While other non-destructive testing (NDT) modalities like visual inspection and ultrasonic testing are also employed, they face similar limitations in terms of efficiency [12], accuracy, and scalability for large-scale automated inspection. This overarching challenge across traditional NDT methods has motivated the exploration of automated solutions using deep learning [13].

With the rapid development of intelligent technologies, deep learning has shown remarkable progress in weld defect detection. Convolutional neural networks (CNNs) have demonstrated the ability to effectively address X-ray-based weld defect detection [14]. Consequently, many researchers have explored this area and achieved promising progress. However, most existing models primarily apply advanced object detection techniques without adequately accounting for the unique characteristics of weld defects. These defects often feature blurred edges, low contrast, and ambiguous patterns, making detection especially difficult in scenarios with complex backgrounds or varying illumination. As a result, conventional deep learning models frequently struggle to deliver sufficient accuracy and robustness in practical industrial applications [15].

To overcome these challenges, this paper proposes WELD-DETR, a defect detection framework based on multi-scale feature fusion and multi-kernel perceptual optimization. The framework incorporates an innovative Hierarchical Feature Pyramid (HFPS) structure, which fuses low-level, high-resolution features with high-level semantic features to significantly improve the detection of micron-level cracks and pores. In addition, the Multi-kernel Perceptual Wavelet Convolution (MPWC) module enhances the model’s sensitivity to edge features and fine textures across multiple scales. Finally, to improve generalization, we construct an industrial-grade welding dataset containing five common defect types and introduce a cross-condition training strategy based on transfer learning, which further strengthens adaptability and performance under diverse working conditions. Experimental results on the self-developed dataset demonstrate that our model achieves excellent detection performance.

The main contributions of this paper are summarized as follows:

(1) We proposed a HFPS Structure, which effectively combines low-level high-resolution features with high-level semantic features. This integration significantly improves the detection of micron-level defects, such as cracks and pores, by leveraging both fine details and semantic context.
(2) We designed a MPWC module, which enhances the model’s ability to capture edge features and fine textures at various scales. This approach enables the model to better recognize defects across different levels of granularity and improves its response to subtle defect patterns.
(3) We constructed and publicly released an industrial-grade X-ray welding dataset covering five common defect types—crack, incomplete penetration, pore, slag inclusion, and incomplete fusion—and introduced a cross-condition training strategy based on transfer learning. Together, these resources enable adaptation to diverse industrial conditions and enhance robustness and generalization, particularly under high noise and strong reflections (see Data Availability Statement for access).

The rest of the paper is organized as follows: Section 2 reviews related research in weld defect detection; Section 3 introduces the dataset used, focusing on the image features of different weld defects and data augmentation methods; Section 4 introduces the model, including the HFPS structure, the MPWC module, and transfer learning; Section 5 details the experimental setup, including environment configuration and evaluation metrics; Section 6 presents and discusses the experimental results, including a comparison with state-of-the-art models; Section 7 concludes with a summary of the key contributions and future work.

## 2. Related Works

### Deep Learning in X-Ray-Based Weld Defect Detection

The application of machine learning in industrial defect detection marks a significant shift from traditional manual inspection to automated analysis [16]. Conventional machine learning methods typically involve three key steps: feature extraction, feature selection, and classification. During the feature extraction phase, various descriptors are commonly used, including grayscale statistical features (mean, variance, entropy, kurtosis), texture features (contrast, correlation, and energy from the Gray-Level Co-occurrence Matrix), geometric features (area, perimeter, circularity of the defect region), and frequency-domain features (wavelet or Fourier transforms) [17,18].

For example, Tian et al. [19] proposed a machine learning-based approach for weld defect detection, integrating induction scanning thermography (IST) with magnetic yoke excitation. Their multifeature fusion method, combining thermal feature images and an artificial neural network (ANN), achieved 99.6% accuracy, reducing false positives in complex backgrounds. Shu et al. [20] introduced a machine learning-based methodology for automatic weld defect classification, using signal processing methods like Short-Time Fourier Transform (STFT) to extract relevant features. By employing deep learning-enhanced classifiers, they achieved up to 96.63% accuracy, highlighting the potential of convolutional neural networks (CNNs) in weld defect detection. Darwish et al. [21] proposed a machine learning framework for laser beam welding, combining supervised and unsupervised learning techniques to analyze electromagnetic emissions and identify defects. This approach achieved 80% accuracy in detecting anomalous welds, showcasing machine learning’s power in interpreting complex welding data.

However, these traditional methods face several fundamental challenges: feature engineering is heavily reliant on domain expertise, requiring different extraction schemes for different welding processes; manually engineered features are sensitive to variations in image quality, leading to performance degradation under noise or poor contrast; and fixed feature descriptors have limited generalization capability when detecting defects with highly variable shapes, such as irregular cracks [22].

With advancements in artificial intelligence, deep learning has significantly transformed the field of X-ray-based weld defect detection [23,24,25]. Convolutional neural network-based approaches, leveraging end-to-end learning, automatically extract discriminative features for defect classification. Architectures such as U-Net, Faster R-CNN, and YOLO have been successfully applied for weld defect localization and classification. For example, Ajmi et al. [26] proposed a deep learning-based method for weld defect classification using a small X-ray dataset, demonstrating that data augmentation and advanced feature extraction techniques improved classification accuracy by 3%. This highlights deep learning’s potential for handling small datasets in industrial defect detection. Yang et al. [27] developed an automatic welding defect localization algorithm based on an improved U-Net network, incorporating data augmentation to enhance defect localization precision. Their model achieved 88.4% accuracy on a public dataset, demonstrating deep learning’s effectiveness for real-time weld defect detection. Zhang et al. [28] introduced a multi-model object detection network for detecting welding defects in pressure pipeline radiographs. By automating defect detection using deep learning techniques, their system significantly enhanced the efficiency and accuracy of weld evaluations. Thi Hoa et al. [29] proposed Weld-CNN, a hybrid convolutional neural network combining sequential convolutional layers with parallel blocks to effectively extract features from X-ray images. Weld-CNN achieved an accuracy of 99.83%, showcasing its potential as a reliable tool for non-destructive weld testing. Zhang et al. [30] developed the DSF-YOLO framework, integrating dynamic staged fusion and multi-scale feature extraction to address challenges such as ambiguous defect boundaries and small defect sizes in X-ray images. Their method improved defect detection accuracy while significantly reducing computational complexity. Zhang et al. [31] also introduced a lightweight model combining DCGAN and MobileNet, optimized for detecting welding defects in imbalanced X-ray datasets. This model achieved an impressive 98.78% accuracy, offering a robust and efficient solution for industrial applications. These studies emphasize the transformative impact of deep learning on X-ray-based weld defect detection, providing enhanced accuracy, efficiency, and scalability compared to traditional methods.

The Detection Transformer (DETR) framework has emerged as a promising end-to-end object detection paradigm, eliminating the need for hand-crafted components like non-maximum suppression. While our work builds upon the real-time optimized RT-DETR [32], several variants have addressed its initial limitations. Deformable DETR [33] replaces global attention with a deformable attention module that attends to a sparse set of key sampling points, significantly accelerating convergence and enhancing small-object detection by leveraging multi-scale features more efficiently. DINO-DETR [34] further strengthens training stability and performance through denoising techniques and improved query formulation. These advancements primarily focus on the decoder structure and training recipe. In contrast, our proposed WELD-DETR introduces core enhancements at the feature level—specifically, the Hierarchical Feature Pyramid Structure (HFPS) and the Multi-kernel Perceptual Wavelet Convolution (MPWC) module. These components are designed to preserve high-resolution spatial details and enrich multi-scale texture representations, which are critical for identifying subtle welding defects. Our architectural contributions are largely orthogonal to the decoder-side improvements in other DETR variants, suggesting potential for future synergy.

However, despite these advancements, existing methods face several limitations that hinder their practical applicability and reliability in industrial settings.

(1) Two-stage detection algorithms, such as Faster R-CNN, achieve high accuracy but are computationally complex, making them unsuitable for real-time detection. On the other hand, single-stage algorithms like YOLO and SSD improve speed but suffer from a significant loss in accuracy when detecting small defects. This trade-off between speed and precision makes it challenging for current methods to meet real-time processing requirements while maintaining high detection accuracy.
(2) High miss detection rate for small defects. Mainstream methods struggle to detect micro-scale defects due to limitations in feature fusion. Traditional Feature Pyramid Networks (FPN) lose fine-grained spatial information during downsampling, making it difficult to detect small defects, such as cracks with a width smaller than 2 pixels. Additionally, background texture interference and noise exacerbate the issue, leading to a higher miss detection rate for small defects.
(3) Adaptability to complex conditions. Existing methods show limited adaptability to challenging industrial conditions. Variations in X-ray imaging equipment, high-noise environments, low-contrast conditions, and strong reflection interference all degrade detection performance. Additionally, changes in welding part position and angle variations can destabilize detection accuracy. These challenges stem from the lack of effective domain adaptation mechanisms and robust feature representation capabilities in current models.

While deep learning has shown tremendous potential in X-ray-based weld defect detection, addressing the challenges of real-time performance, small defect detection, and adaptability to industrial conditions is critical for the widespread application of these technologies in high-stakes industrial environments. Advancing these areas of research will be key to achieving reliable, robust, and scalable solutions for automated welding inspection.

## 3. Dataset

### 3.1. Experimental Dataset

**Figure 1:**
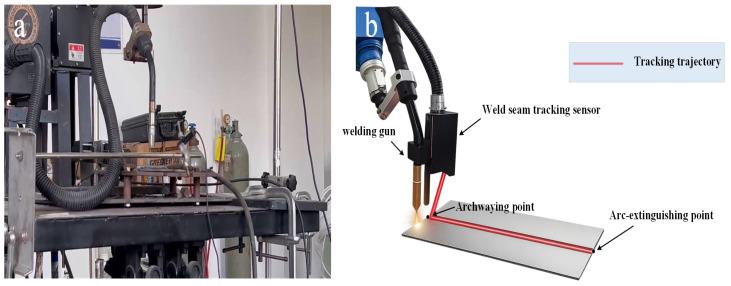
Experimental collection equipment. (**a**) Model of the robotic welding equipment; (**b**) Physical setup with key components: welding gun, tracking sensor, and key points.

To verify the effectiveness of the proposed WELD-DETR framework, we constructed an industrial-grade X-ray dataset specifically designed for weld defect detection. The dataset was collected using an industrial X-ray imaging system, ensuring a high level of reliability and realism in the data. As illustrated in Figure 1, the imaging system utilized a high-resolution digital radiography unit, which is capable of capturing intricate internal images of welded specimens without compromising their structural integrity. The system was calibrated with the following parameters: tube voltage of 60–90 kV, current of 3.0–5.0 mA, and exposure time of 0.8–1.5 s. All weld specimens were fabricated from carbon steel (Q235 grade) with thickness ranging from 8–15 mm, using gas metal arc welding (GMAW) process. The source-to-detector distance was maintained at 800 mm with a magnification factor of 1.8. Imaging was performed in a controlled environment at 23 ± 2 °C with 50 ± 5% relative humidity. The system was meticulously calibrated to operate under consistent parameters, including X-ray energy, exposure time, and detector sensitivity, ensuring that each image was of uniform quality across all samples. This standardization of imaging conditions allowed for reproducible and comparable results, minimizing any variations that could affect the detection performance.

To ensure the integrity and reliability of the dataset, we used an internal annotation platform to label the X-ray images with bounding boxes for five defect classes—crack, incomplete penetration, pore, slag inclusion, and incomplete fusion. Before annotation, we established unified criteria and operating guidelines (defect definitions, typical appearances, minimum box size, and rules for handling overlapping or adjacent defects). Each image was first annotated by an inspector with experience in radiographic weld inspection and then independently reviewed and revised by a second experienced annotator. Afterward, we ran script-based quality checks (label validity, coordinate bounds, zero-area/duplicate boxes) and conducted sampled manual audits for easily confused classes. To improve computational efficiency and model training stability, all images were resized to a uniform 640 × 640 resolution. Special care was taken to preserve key defect details, ensuring important information was not lost while meeting processing efficiency requirements.

**Figure 2:**
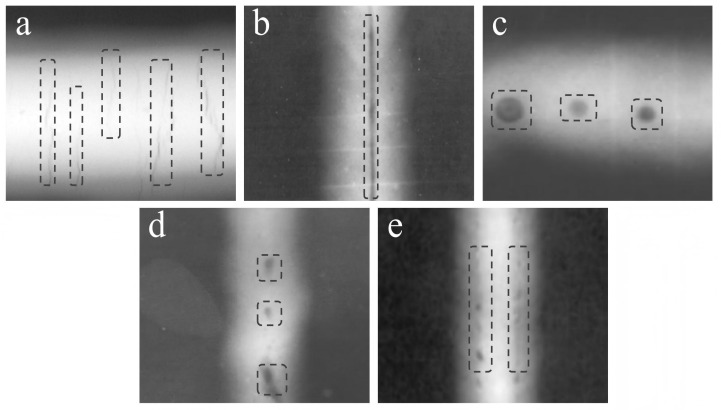
X-ray images of the weld defect. (**a**) Crack; (**b**) Incomplete penetration; (**c**) Pore; (**d**) Slag inclusion; (**e**) Incomplete fusion.

As illustrated in Figure 2, the dataset covers five representative categories of weld defects: Crack, Incomplete penetration, Pore, Slag inclusion, and Incomplete fusion. These defects represent the most critical quality issues in industrial welding. Table 1 summarizes the distribution and characteristics of these defects, including their radiographic appearance and relative proportions within the dataset. In total, the dataset contains 3247 high-resolution X-ray images, each with one or more annotated defect instances, amounting to 5892 labeled defect regions.

To facilitate model training and evaluation, the entire dataset was randomly partitioned into training, validation, and test sets with a ratio of 70:15:15. Stratified sampling was employed to ensure that the distribution of all five defect categories remained consistent across each split. Consequently, the training set contains 2273 images, while both the validation and test sets comprise 487 images each. This partitioning strategy guarantees a fair and reproducible assessment of the model’s performance.

The five defect categories were selected due to their high industrial prevalence, critical impact on structural safety, and diverse morphological characteristics in X-ray images, ensuring comprehensive coverage of common arc welding quality issues.

### 3.2. Data Augmentation

To address the issues of limited dataset size and uneven sample distribution, this paper introduces a systematic data augmentation scheme to expand the diversity of training samples, thereby improving the model’s generalization ability and resistance to overfitting.

**Figure 3:**
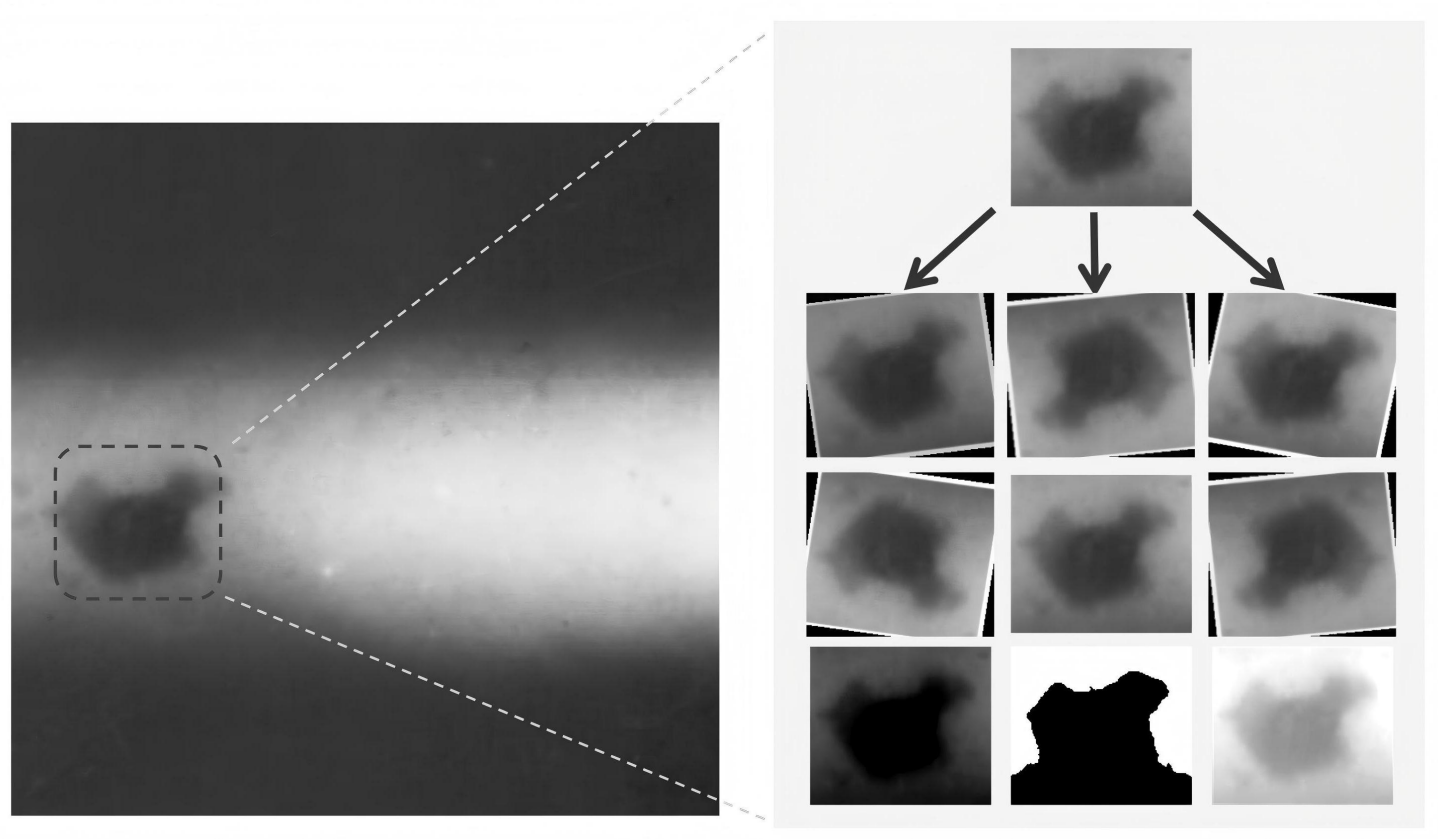
Data augmentation for welding defect.

As illustrated in Figure 3, our data augmentation strategy encompasses geometric transformations, pixel-level transformations, and a progressive augmentation approach.

Geometric transformations simulate various shooting angles and distances through random rotation (with an angle range from −12° to +12°) and random flipping operations (with a horizontal flip probability set at 0.6 and a vertical flip probability set at 0.2), further enhancing the model’s adaptability to different object orientations.

Pixel-level transformations employ a random perturbation strategy in color space: random adjustments to image brightness within ±25%, contrast adjustments within the range of 0.75 to 1.25 times, and saturation variations between 0.8 and 1.3 times. Additionally, random Gaussian blur (with a kernel size between 1 and 3 pixels) is introduced to enhance the model’s resistance to noise.

To optimize the augmentation effects, a progressive augmentation strategy is designed: in the early stages of training, milder transformation parameters are used to ensure stable convergence, and the transformation strength gradually increases according to a sigmoid scheduling function strength=1/(1+exp(−epoch)) as the training progresses.

With this augmentation scheme, the dataset is expanded from 3247 images to 12,000 images. This specific augmentation scale was empirically determined through systematic experiments evaluating model performance across different dataset sizes, with 12,000 images found to achieve the optimal trade-off between feature diversity and computational efficiency.

## 4. Methods

### 4.1. Overall Architecture of WELD-DETR

**Figure 4:**
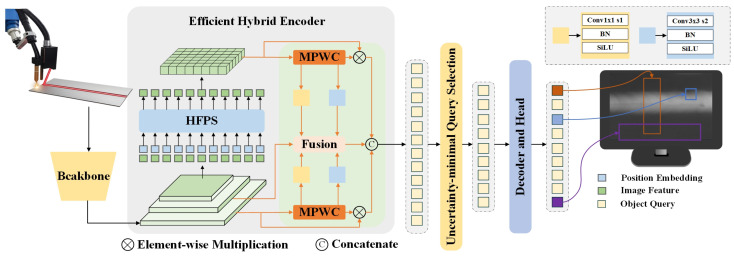
WELD-DETR overall architecture.

WELD-DETR was a welding-defect detection model inspired by the RT-DETR architecture [35]. By combining multi-level features with multi-scale perceptual capability, it enabled real-time, precise detection of micrometer-level defects during welding, such as cracks, porosity, and slag inclusions. As illustrated in Figure 4, the WELD-DETR architecture consisted of four main components: a feature-extraction backbone, an HFPS structure, an MPWC module, and a decoder with prediction heads.

The input to WELD-DETR was a welding X-ray image of size $X\in R^{3\times 640\times 640}$. After processing through the backbone network, feature maps at three different scales were generated: $F_{1}\in R^{256\times 160\times 160}$, $F_{2}\in R^{512\times 80\times 80}$, and $F_{3}\in R^{1024\times 40\times 40}$. First, WELD-DETR passed input welding images through the backbone network for initial feature extraction, producing fundamental low-level features such as edges and textures. Next, the HFPS structure processed these features, combining high-resolution low-level and high-level semantic features. Low-level features preserved fine details (e.g., cracks or pores), while high-level features provided global context. The fusion of these features enhanced the model’s ability to detect subtle defects.

To further improve sensitivity to details, WELD-DETR introduced the MPWC module. MPWC used multi-scale convolution operations to extract edge and texture cues, particularly for minute defect details like subtle crack variations or surface texture changes. After HFPS structure and MPWC module processing, features from different levels were merged for further processing. The model then employed an Uncertainty-minimal Query Selection strategy to focus on the most relevant image regions, maximizing certainty about defect locations and improving detection precision. This strategy utilized entropy-based uncertainty measures, calculating predictive entropy from the decoder’s output distributions to identify and prioritize queries with minimal uncertainty. By focusing computational resources on the most reliable predictions, this approach enhanced both efficiency and detection accuracy.

Finally, the fused features were input into the decoder, which extracted defect-related information through an iterative attention mechanism. The model used Multi-head Attention and Multi-scale Deformable Attention to capture contextual information, effectively extracting key cues related to defect positions and categories. The prediction heads then output bounding boxes and class probabilities, achieving accurate defect localization and classification.

Additionally, WELD-DETR included an industrial-grade welding dataset with five common defect types. To enhance generalization across different industrial settings, the model used a cross-condition training strategy and transfer learning, enabling it to adapt to environmental variations and maintain high detection accuracy even under challenging conditions.

### 4.2. HFPS Structure

To address the multi-scale target recognition challenges in X-ray welding defect detection, particularly the detection difficulties of micrometer-level defects (such as fine cracks and pinholes) in low-contrast X-ray images, this paper proposed an innovative HFPS structure. This structure aimed to resolve critical technical bottlenecks in traditional feature pyramid networks when processing welding defect detection tasks: the loss of minute defect information due to down-sampling operations, the inability of single-scale feature extraction to balance local details with global context, and insufficient feature discrimination capability in the low signal-to-noise ratio environment characteristic of X-ray images.

**Figure 5:**
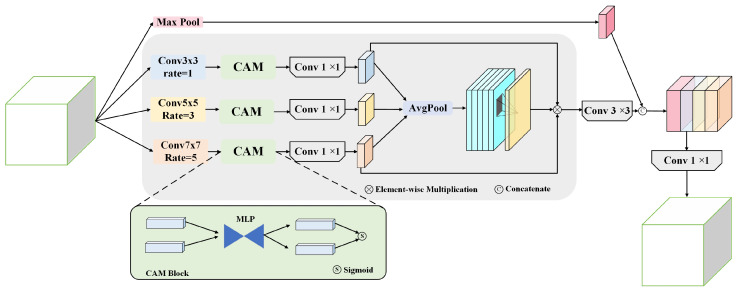
HFPS Structure.

As illustrated in Figure 5, the HFPS structure took multi-scale backbone features as inputs: $F_{1}\in R^{256\times 160\times 160}$, $F_{2}\in R^{512\times 80\times 80}$, and $F_{3}\in R^{1024\times 40\times 40}$. The core architecture of HFPS structure adopted a three-branch parallel design, with each branch specifically optimized for different scales of defect features. The first branch employed a 3×3 convolution kernel (dilation rate r=1) to focus on capturing local detail features, effectively identifying edge information of fine cracks with widths ranging from 0.05–0.2 mm; the second branch used a 5×5 convolution kernel (dilation rate r=3) to obtain medium receptive fields, suitable for detecting medium-sized pores and inclusions with diameters of 0.2–1.0 mm; the third branch configured a 7×7 convolution kernel (dilation rate r=5) to construct large-scale contextual receptive fields for understanding overall weld structure and spatial distribution patterns of large defects.

The mathematical expression for this multi-branch design was:

(1) $$F_{i}={\mathrm{Conv}}_{k_{i}\times k_{i}}^{r_{i}}\left(X\right)$$

where $\left(k_{1},r_{1}\right)=\left(3,1\right)$, $\left(k_{2},r_{2}\right)=\left(5,3\right)$, $\left(k_{3},r_{3}\right)=\left(7,5\right)$, and $X\in R^{H\times W\times C}$ represented the input feature map. Each branch processed its respective input feature map and outputted a feature map with 256 channels: the first branch produced $F_{1}^{\prime }\in R^{256\times 160\times 160}$, the second outputted $F_{2}^{\prime }\in R^{256\times 80\times 80}$, and the third generated $F_{3}^{\prime }\in R^{256\times 40\times 40}$.

To address the characteristics of weak defect signals and severe background noise interference in X-ray images, each feature extraction branch integrated a Channel Attention Module (CAM) that achieved adaptive feature enhancement by learning the importance weights of different feature channels for defect detection tasks. The CAM implementation mechanism first compressed spatial dimensions through a global average pooling operation:

(2) $$\mathrm{GAP}\left(F\right)=\frac{1}{HW}\sum_{i=1}^{H}\sum_{j=1}^{W}F_{i,j}$$

to obtain global statistical information for each channel, then employed a two-layer multi-layer perceptron to learn nonlinear dependencies between channels, where the first layer used a ReLU activation function for dimensionality reduction and the second layer generated attention weights through the Sigmoid function. Finally, the learned channel weights were element-wise multiplied with the original feature map to achieve feature recalibration, with the mathematical expression:

(3) CAM(F)=F⊙σ(MLP(ReLU(MLP(GAP(F)))))

where ⊙ denoted element-wise multiplication.

The innovative core of HFPS lay in its hierarchical feature fusion strategy, which effectively balanced the detail preservation capability and semantic understanding capability of feature representation. In the specific implementation process, outputs from the three branches were first unified in channel dimensions through 1×1 convolutions to generate feature maps with identical channel numbers, ensuring dimensional consistency for subsequent fusion operations. Simultaneously, the input feature map underwent max pooling operations to extract salient features, which were then concatenated with the three-branch features to form a multi-scale feature set. The max pooling operation could highlight peak responses in defect regions, contributing to enhanced sensitivity in defect detection.

Subsequently, average pooling operations are applied to the concatenated features for spatial dimension compression to generate global feature descriptors, which effectively integrate spatial distribution information of multi-scale features and provide global context for subsequent attention weight computation. Finally, spatial attention-guided feature fusion is achieved through the operation:

(4) $$F_{\mathrm{att}}=F_{\mathrm{global}}⊙{\mathrm{Conv}}_{3\times 3}\left(\mathrm{Concat}\left(F_{1}^{\prime },F_{2}^{\prime },F_{3}^{\prime }\right)\right)$$

where the concatenated feature $\mathrm{Concat}(F_{1}^{\prime },F_{2}^{\prime },F_{3}^{\prime })$ has dimension $R^{768\times 80\times 80}$, and the 3×3 convolution is responsible for learning spatial correlations among multi-scale features to generate spatial weight maps that guide the model to focus on potential defect regions.

The feature fusion mechanism of our HFPS module is formally described in Algorithm 1. HFPS structure demonstrates technical advantages compared to traditional feature pyramid networks: enabling fine cracks below 0.05 mm to maintain detectable signal strength in feature maps; the introduction of dilated convolutions expands the receptive field range without increasing parameter count, capable of simultaneously perceiving local texture changes and global structural patterns, making the model more robust when detecting irregularly shaped defects; the dual attention mechanism achieves adaptive feature selection and enhancement, where channel attention highlights feature dimensions sensitive to defects and spatial attention focuses on key regions where defects may appear, with their synergistic effect significantly improving signal-to-noise ratio; despite employing a multi-branch design, through parameter sharing and progressive fusion strategies, a good balance between accuracy and efficiency is achieved.


**Algorithm 1:** Hierarchical Feature Pyramid Structure (HFPS).
**Require:** Multi-scale backbone features: $F_{1},F_{2},F_{3}$**Ensure:** Fused multi-scale feature $F_{out}$ 1: **Parallel Multi-branch Processing:** 2: **for** i=1 to 3 **do** 3:    $F_{i}^{\prime }\leftarrow {\mathrm{Conv}}_{k_{i}\times k_{i},r_{i}}\left(F_{i}\right)$ {Multi-branch convolution with kernel $k_{i}$ dilation $r_{i}$} 4:    $F_{i}^{\prime }\leftarrow \mathrm{CAM}\left(F_{i}^{\prime }\right)$ {Channel Attention Module} 5: **end for** 6:  7: **Feature Fusion:** 8: $F_{concat}\leftarrow \mathrm{Concat}\left(F_{1}^{\prime },\mathrm{UpSample}\left(F_{2}^{\prime }\right),\mathrm{UpSample}\left(F_{3}^{\prime }\right)\right)$ 9: $F_{global}\leftarrow \mathrm{GlobalAvgPool}\left(F_{concat}\right)$10: $W_{spatial}\leftarrow \mathrm{Sigmoid}\left({\mathrm{Conv}}_{3\times 3}\left(F_{concat}\right)\right)$ {Spatial attention weights}11: $F_{att}\leftarrow F_{concat}⊙W_{spatial}$12: 13: **Output:**14: $F_{out}\leftarrow {\mathrm{Conv}}_{1\times 1}\left(F_{att}\right)$ {Channel reduction}15: 16: **return** $F_{out}$



### 4.3. MPWC Module

**Figure 6:**
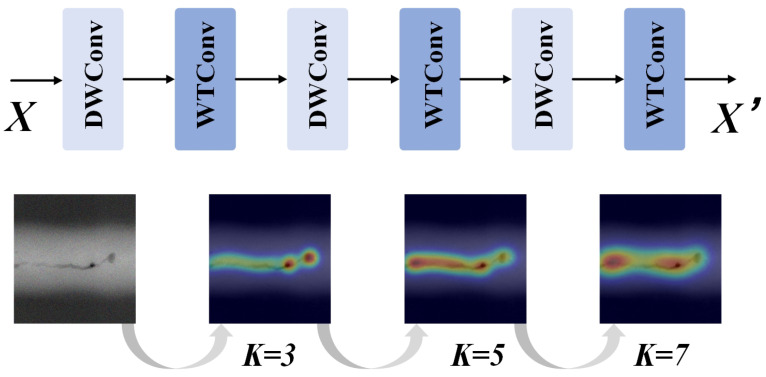
MPWC Module.

**Figure 7:**
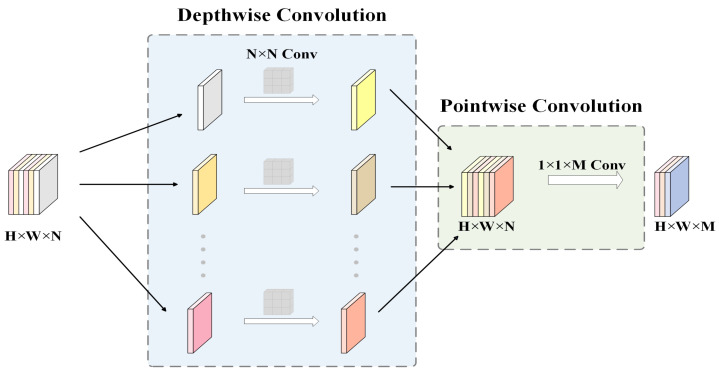
DWConv Unit.

**Figure 8:**
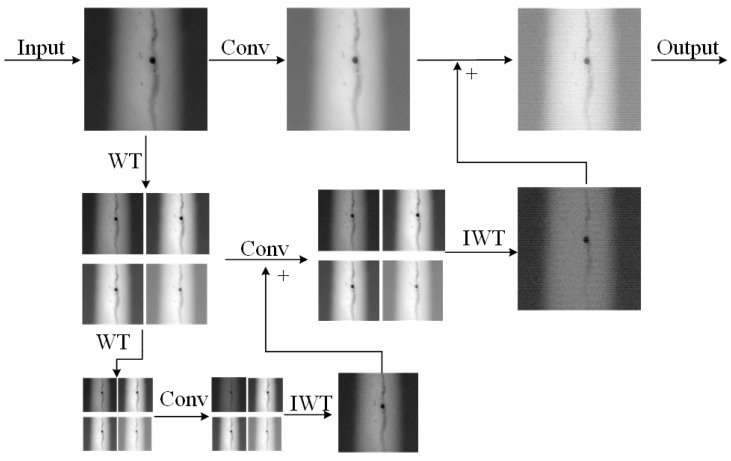
WTConv Unit.

In response to the challenges of multi-scale feature extraction, detail capture, and computational efficiency in X-ray image defect detection, this paper proposed an innovative MPWC module, as illustrated in Figure 6. Defects in X-ray images often exhibited varying scales, shapes, and texture features, which were often subtle and similar to the background textures. Traditional Convolutional Neural Networks, due to their fixed receptive fields and localized feature extraction mechanisms, struggled to capture multi-level, multi-scale critical information, leading to low detection precision and issues like false negatives or positives. To address this, the MPWC module integrated Depthwise Convolution (DWConv, Figure 7) and Wavelet Transform Convolution (WTConv, Figure 8) [36,37] as its core sub-modules. This design enabled multi-scale feature extraction concurrently in the spatial and frequency domains, thereby enhancing the model’s capability to detect defects of diverse scales and types.

Specifically, the DWConv sub-module extracts low-level spatial features from the input image $X\in R^{H\times W\times C}$, capturing edges, contours, and other fine details crucial for initial defect localization. Given this input feature map, the DWConv sub-module maintains the spatial dimensions while processing each channel independently, producing $Y_{\mathrm{DW}}\in R^{H\times W\times C}$. The DWConv operation can be expressed as:

(5) $$Y_{\mathrm{DW}}=X*W_{\mathrm{DW}},$$

where *X* is the input image, $W_{\mathrm{DW}}\in R^{K\times K\times C}$ is the depthwise convolution kernel, and $Y_{\mathrm{DW}}\in R^{H\times W\times C}$ is the output feature map. The advantage of DWConv is its independent convolution across each input channel, which reduces computational cost while better preserving channel independence, making it particularly effective at extracting local features. After DWConv, the feature map $Y_{\mathrm{DW}}$ is passed to the WTConv sub-module, where wavelet transform is applied to extract frequency features at multiple scales, helping the model to capture texture and detail, especially for detecting blurry or small defects. The wavelet transform operation can be expressed as:

(6) $$Y_{\mathrm{WT}}=\mathrm{WT}\left(Y_{\mathrm{DW}},K\right),$$

where the input $Y_{\mathrm{DW}}\in R^{H\times W\times C}$ undergoes wavelet transformation at scale *K*, producing output $Y_{\mathrm{WT}}\in R^{H\times W\times C}$ with enhanced frequency-domain features. In our implementation, the Haar wavelet was selected as the wavelet function WT due to its computational efficiency and strong edge detection capability, which aligns well with the sharp intensity transitions characteristic of weld defects. By choosing multiple scales K=3,5,7, WTConv can extract detail information at different frequency ranges, significantly improving the model’s ability to recognize defects at various scales.

The MPWC module alternates between DWConv and WTConv, not only extracting low-frequency spatial information in the spatial domain but also enhancing the details through wavelet transform in the frequency domain. This allows the model to perceive the image at multiple scales. The final output feature map $X^{\prime }$ can be expressed as:

(7) $$X^{\prime }=F\left(X,\left\{W_{\mathrm{DW}},W_{\mathrm{WT}}\right\}\right),$$

where the composite operation F transforms the input $X\in R^{H\times W\times C}$ to output $X^{\prime }\in R^{H\times W\times C}$, preserving spatial dimensions while enhancing multi-scale features. DWConv focuses on extracting spatial features, while WTConv extracts frequency features at multiple scales, and their combination effectively enhances the model’s sensitivity to details and texture. This multi-scale feature extraction not only handles defects of different scales and shapes in the image but also improves the model’s ability to capture fine details in complex backgrounds, especially for small cracks, pores, and other subtle defects.

The feature fusion mechanism of our MPWC module is formally described in Algorithm 2. The MPWC module not only improved defect detection accuracy but also optimized computational efficiency. By utilizing DWConv, the module significantly reduced computational complexity. Unlike traditional convolutions, where each filter was applied across all input channels, DWConv operated independently on each channel, drastically lowering the number of parameters and operations required. This approach maintained essential spatial relationships while reducing computational overhead, enabling the module to process high-dimensional X-ray images more efficiently. Additionally, the wavelet transform in WTConv captured multi-scale frequency-domain features without adding significant computational cost. The wavelet transform decomposed the image into different frequency components, allowing the model to extract both fine details and broader structural information. WTConv leveraged this process efficiently, focusing on relevant image features across multiple scales while minimizing overhead. By combining depthwise convolutions with wavelet transforms, the MPWC module struck a balance between high performance and computational efficiency, making it suitable for real-time defect detection in environments where both accuracy and speed were critical.
**Algorithm 2:** Multi-kernel Perceptual Wavelet Convolution (MPWC).**Require:** Input feature map $X\in R^{H\times W\times C}$**Ensure:** Enhanced feature map $X^{\prime }\in R^{H\times W\times C}$ 1: 2: **Spatial Feature Extraction:** 3: $Y_{DW}\leftarrow X*W_{DW}$ {Depthwise convolution for spatial features} 4: 5: **Frequency Feature Extraction:** 6: $Y_{WT}\leftarrow \mathrm{WT}\left(Y_{DW},K\right)$ {Wavelet transform at scale *K*} 7: $Y_{WT}\leftarrow {\mathrm{Conv}}_{1\times 1}\left(Y_{WT}\right)$ {Feature transformation} 8: $Y_{WT}\leftarrow \mathrm{IWT}\left(Y_{WT}\right)$ {Inverse wavelet transform} 9: 10: **Feature Fusion:**11: $X^{\prime }\leftarrow Y_{DW}+Y_{WT}$ Residual connection12: **return** $X^{\prime }$

### 4.4. Transfer Learning

To enhance the generalization capability and robustness of the WELD-DETR model in complex and variable industrial environments, this paper proposes a cross-condition training strategy based on transfer learning [38]. Traditional deep learning methods typically assume that training data and test data follow the same distribution. However, in practical industrial applications, welding defect detection faces numerous challenges: different X-ray imaging equipment parameters, varying illumination conditions, welded components of different materials, and complex noise interference all contribute to significant data distribution differences. This distribution shift problem severely affects the detection performance of models in actual deployment, causing models that perform excellently in laboratory environments to potentially experience dramatic performance degradation in real industrial scenarios. To address these challenges, a multi-stage progressive transfer learning framework is designed that fully utilizes the general visual feature extraction capabilities of pre-trained models and gradually adapts to the specific task requirements of welding defect detection through domain adaptation techniques. The entire transfer learning process is structured as three interconnected stages, each with clear objectives and optimization strategies.

**Pre-training Stage**: This stage aims to establish a strong foundation for general visual feature extraction. We first pre-train the backbone network of the model on large-scale general visual datasets (such as ImageNet), enabling it to learn rich low-level visual features, including edges, textures, shapes, and other basic visual elements. The objective function for this stage can be expressed as:

(8) $$\theta_{pre}=arg\;\underset{\theta }{min}L_{pre}\left(\theta \right)=\frac{1}{N_{pre}}\sum_{i=1}^{N_{pre}}\ell \left(f_{\theta }\left(x_{pre}^{i}\right),y_{pre}^{i}\right)$$

where $\theta_{pre}$ represents the model parameters obtained through pre-training, $f_{\theta }$ represents the neural network function, and *ℓ* is the loss function. Through large-scale pre-training, the model acquires sensitivity to various visual patterns, laying a foundation for subsequent domain-specific learning.

**Domain Adaptation Stage**: This is the core stage of transfer learning, aimed at transferring general visual feature representation capabilities to the specific domain of welding defect detection. Given source domain dataset $D_{s}={\left\{\left(x_{s}^{i},y_{s}^{i}\right)\right\}}_{i=1}^{N_{s}}$ and target domain dataset $D_{t}={\left\{\left(x_{t}^{j},y_{t}^{j}\right)\right\}}_{j=1}^{N_{t}}$, we adopt a joint optimization strategy that simultaneously considers the preservation of source domain knowledge and the learning of target domain features. The loss function for domain adaptation is designed as:

(9) $$L_{adapt}=\lambda_{s}L_{s}\left(\theta \right)+\lambda_{t}L_{t}\left(\theta \right)+\lambda_{reg}R\left(\theta \right)$$

where $L_{s}\left(\theta \right)=\frac{1}{N_{s}}\sum_{i=1}^{N_{s}}\ell \left(f_{\theta }\left(x_{s}^{i}\right),y_{s}^{i}\right)$ is the source domain loss, ensuring that the model maintains the general feature extraction capabilities learned in the source domain; $L_{t}\left(\theta \right)=\frac{1}{N_{t}}\sum_{j=1}^{N_{t}}\ell \left(f_{\theta }\left(x_{t}^{j}\right),y_{t}^{j}\right)$ is the target domain loss, prompting the model to adapt to the specific requirements of welding defect detection; R(θ) is the regularization term, preventing overfitting and promoting smooth knowledge transfer. The weight coefficients $\lambda_{s}$, $\lambda_{t}$, and $\lambda_{reg}$ are determined through experimental tuning to balance the contributions of different loss terms.

To better handle distribution differences between domains, an adversarial training mechanism was introduced. By introducing a domain discriminator $D_{domain}$, the model learns to generate domain-invariant feature representations:

(10) $$L_{adversarial}=E_{x∼D_{t}}\left[\log D_{domain}\left(f_{\theta }\left(x\right)\right)\right]+E_{x∼D_{s}}\left[log\;\left(1-D_{domain}\left(f_{\theta }\left(x\right)\right)\right)\right]$$

This adversarial training strategy forces the feature extractor to generate features that cannot be distinguished by the domain discriminator, thereby improving the model’s generalization capability under different industrial conditions.

**Fine-tuning Stage**: After obtaining domain-adaptive foundation features, we enter the fine-grained optimization stage specifically for welding defect detection tasks. Considering the imbalanced distribution characteristics of five defect types (crack, incomplete penetration, pore, slag inclusion, incomplete fusion) in the welding defect dataset, a class weighting strategy was adopted to ensure the model had good detection capabilities for all defect types:

(11) $$L_{balanced}=\sum_{c=1}^{5}w_{c}\cdot L_{c}$$

where the weight $w_{c}=\frac{N_{total}}{5\cdot N_{c}}$, with $N_{total}$ being the total number of samples and $N_{c}$ being the number of samples for the *c*-th defect type. This weighting strategy effectively alleviates the data imbalance problem and improves the model’s detection performance for minority class defects. Beyond the explicit class weighting in Equation (11), our architecture inherently mitigates imbalance. The HFPS preserves crucial details for rare, small defects, while the MPWC enhances general texture sensitivity. This prevents the feature extractor from biasing towards majority classes and improves detection robustness across all categories. To adapt to dynamic changes during the training process, we employ an adaptive learning rate adjustment strategy:

(12) $$\eta_{t}=\eta_{0}\cdot {\left(1-\frac{t}{T}\right)}^{\alpha }\cdot \left(1+\cos\left(\frac{\pi t}{T}\right)\right)$$

where $\eta_{0}$ is the initial learning rate, *t* is the current training epoch, *T* is the total number of training epochs, and α is the decay exponent. This learning rate scheduling strategy combines the advantages of polynomial decay and cosine annealing, maintaining a high learning rate in the early training stage for rapid adaptation to new domains, and using a smaller learning rate in the later stage for fine-tuning.

To further enhance the model’s robustness to different imaging conditions, we design a multi-condition perception mechanism. By introducing a condition encoder $E_{cond}$, the model can explicitly model feature variations under different working conditions:

(13) $$\begin{array}{cc} z_{cond} & =E_{cond}\left(c\right) \end{array}$$

(14) $$\begin{array}{cc} f_{adapted}\left(x\right) & =f_{\theta }\left(x\right)+\gamma \cdot z_{cond} \end{array}$$

where *c* represents the condition vector containing imaging parameters, environmental noise levels, and other information, and γ is the adjustment factor.

Integrating all components, the final training objective function is:

(15) $$L_{total}=L_{adapt}+\beta L_{adversarial}+\delta L_{condition}$$

where β and δ are the weight coefficients for adversarial loss and condition loss, respectively, optimized through methods such as grid search.

The advantages of this progressive transfer learning strategy include: fully utilizing the general feature extraction capabilities of pre-trained models to accelerate model convergence; effectively handling distribution shift problems through domain adaptation techniques; improving model robustness in complex industrial environments through class balancing and multi-condition perception mechanisms.

### 4.5. Implementation Details of Multi-Condition Mechanism

This subsection elaborates on the practical implementation of the multi-condition perception mechanism, detailing the composition of the condition vector and its operational use during inference. The condition vector c is designed to encode a concise signature of the imaging environment, integrating several key operational parameters. It incorporates a quantitative measure of image noise, estimated from the standard deviation within a homogeneous background region; the global image contrast, computed directly from the input data; and a categorical identifier representing the specific X-ray equipment in use. These parameters are normalized and concatenated to form a unified condition vector.

During inference, the system dynamically computes the noise and contrast descriptors directly from each input X-ray image. The equipment identifier is set to a default value that corresponds to the primary imaging setup characterized during the training phase, serving as a robust prior in the absence of specific source information. This assembled condition vector is then processed by the condition encoder $E_{\mathrm{cond}}$, which is implemented as a compact multi-layer perceptron. The output of this encoder, the conditioning feature $z_{\mathrm{cond}}$, is used to adaptively modulate the primary feature map via the feature-wise transformation described in Equation (14). This process enables the model to dynamically adjust its feature representations in response to the specific operational context of the input data, enhancing robustness across varying imaging conditions.

## 5. Experiments

### 5.1. Experimental Environment

To assess the performance, generalization capabilities, and efficiency of the proposed WELD-DETR model, a comprehensive series of quantitative and qualitative experiments were conducted. These experiments were designed to evaluate the model against a variety of baseline approaches [39].

As illustrated in Table 2, the experiments were carried out on a machine running Windows 10 (64-bit), equipped with 32 GB of RAM, an NVIDIA GeForce RTX 2060 GPU (NVIDIA Corporation, Santa Clara, CA, USA), and an Intel(R) Core(TM) i7-10870H CPU @ 2.20 GHz (Intel Corporation, Santa Clara, CA, USA). The software environment consisted of Python 3.8.5, with PyTorch version 1.10.0 and CUDA 11.3 utilized for GPU acceleration.

### 5.2. Hyperparameter Settings

As illustrated in Table 3, all experiments were executed under the same conditions to ensure consistency. The images used for training were resized to a resolution of 640 × 640 pixels, and a batch size of 16 was employed during training. The initial learning rate was set to 0.01, and the optimizer used was Stochastic Gradient Descent (SGD) with a momentum of 0.937. The training process was carried out over a total of 200 epochs.

### 5.3. Evaluation Metrics

The evaluation of the model’s performance was carried out using a set of key metrics, which include Precision (P), Recall (R), Mean Average Precision (mAP), Floating Point Operations (FLOPs), Frames Per Second (FPS), and the total number of model parameters (M). The definitions for these metrics are as follows:

(16) $$P=\frac{\mathrm{TP}}{\mathrm{TP}+\mathrm{FP}},$$

where true positive (TP) represents the number of actual positive samples correctly predicted as positive, while false positive (FP) refers to the number of actual negative samples incorrectly predicted as positive.

(17) $$R=\frac{\mathrm{TP}}{\mathrm{TP}+\mathrm{FN}},$$

In the formula, false negative (FN) indicates the number of actual positive samples predicted as negative.

(18) $$\mathrm{mAP}=\frac{1}{N}\sum_{i=1}^{N}{\mathrm{AP}}_{i},$$

where average precision (AP) measures the average precision for a specific class of targets at various recall points and corresponds to the area under the precision–recall (PR) curve. When the Intersection over Union (IoU) threshold is set to 0.5, AP is specifically denoted as AP50.

(19) $$\mathrm{FPS}=\frac{1}{T_{\mathrm{inference}}},$$

where $T_{\mathrm{inference}}$ represents the processing time per image, encompassing the complete inference pipeline after images are resized to the standard input resolution of 640×640 pixels. This provides a consistent measure of the model’s real-time detection capability under its intended operational configuration.

To ensure the robustness and statistical significance of our results, all experiments were conducted with five different random seeds. The reported performance metrics (Precision, Recall, mAP) are presented as the mean ± standard deviation across these independent runs. This approach provides a measure of the variability and reliability of our model’s performance.

## 6. Results and Discussion

### 6.1. Comparison of Detection Performance

To validate the effectiveness of the proposed WELD-DETR model, we conducted a comprehensive comparison with several state-of-the-art object detection methods on an industrial-grade welding defect dataset. As illustrated in Table 4, the compared methods include general deep learning object detection techniques such as Faster R-CNN, Mask R-CNN, SSD, EfficientDet-D0, MobileNetV3-SSD, and RetinaNet, as well as recent advanced detection models like YOLOv5n, YOLOv8s, YOLOv8n, YOLOv10s, and RT-DETR-R18. These methods encompass both traditional two-stage detection approaches and modern one-stage paradigms, providing a broad benchmark for evaluation. To ensure a fair and reproducible comparison, all models were implemented using the PyTorch framework on the same experimental setup with an Intel Core i7-10870H CPU @ 2.20 GHz, 32 GB RAM, and an NVIDIA RTX 2060 GPU.

The experimental results demonstrate that WELD-DETR outperforms all the SOTA methods across various evaluation metrics. WELD-DETR achieved an impressive mAP of 98.2 ± 0.1% at IoU thresholds of 0.5 to 0.95, ranking first among all methods, indicating its superior and stable overall detection performance across different IoU thresholds. The model also achieved 96.8 ± 0.1% precision and 94.5 ± 0.2% recall, effectively minimizing both false positives and false negatives while accurately identifying welding defects. Despite its excellent detection accuracy, WELD-DETR maintained a remarkable inference speed of 58.0 FPS on the RTX 2060 platform, showcasing an exceptional balance between detection accuracy and computational efficiency.

In comparison to mainstream detection methods, WELD-DETR demonstrates significant improvements across all metrics. It notably outperforms YOLOv10s (mAP: 94.5%, precision: 93.1%, recall: 91.4%) by 3.7%, 3.7%, and 3.1%, respectively, and surpasses RT-DETR-R18 (mAP: 92.6%, precision: 93.5%, recall: 93.8%) by 6.4%, 1.3%, and 0.7%. More remarkably, WELD-DETR achieves substantial gains over traditional methods, with a 16.2% improvement over Faster R-CNN, a 22.2% improvement over SSD, and a 14.7% improvement over EfficientDet-D0 in terms of mAP. The model also demonstrates superior performance compared to recent YOLO variants, improving mAP@0.5–0.95 by 7.4% over YOLOv8s, 12.7% over YOLOv8n. Notably, these performance advantages are statistically significant and consistent across all five experimental runs, as evidenced by the low standard deviations reported in Table 4.

The significant performance improvement can be attributed to three key architectural innovations that synergistically enhance detection capability. First, the HFPS Structure achieves more effective multi-scale feature extraction and fusion compared to traditional Feature Pyramid Networks. This design strategically combines high-resolution spatial information from lower-level features with rich semantic context from higher-level features, creating a comprehensive representation that excels in detecting fine-scale defects such as minute cracks and tiny pores. The effectiveness of HFPS is particularly evident when comparing WELD-DETR with YOLOv8s (mAP: 90.8%) and EfficientDet-D0 (mAP: 83.5%), resulting in improvements of 7.4% and 14.7%, respectively. Second, the MPWC module significantly enhances the model’s ability to preserve fine-grained texture information while improving edge feature recognition. This module uses multi-kernel wavelet transforms to maintain critical spatial–frequency domain information, enabling superior detection of subtle defect patterns often missed by traditional convolution methods. The effectiveness of MPWC is reflected in the substantial precision improvement over conventional CNN methods like Faster R-CNN (88.5% compared with 96.8%) and Mask R-CNN (87.9% compared with 96.8%), demonstrating its ability to reduce false positives while maintaining high sensitivity to actual defects.

From a computational efficiency perspective, WELD-DETR strikes an optimal balance between model complexity and performance. With 15.6 M parameters, the model achieves exceptional accuracy while maintaining reasonable computational demands. Although its parameter count exceeds that of ultra-light models like YOLOv5n (3.8 M) and YOLOv8n (3.2 M), the significant accuracy gains justify this increase. Furthermore, compared to RT-DETR-R18 (19.5 M parameters), which has similar complexity, WELD-DETR outperforms it in detection performance with 20% fewer parameters, demonstrating the efficiency of the proposed architecture.

The experimental evaluation highlights several key advantages of WELD-DETR for industrial welding quality control applications. The model’s ultra-high recall rate of 94.5 ± 0.2% significantly reduces the risk of missing critical defects, which is crucial in safety-critical applications where undetected defects could lead to catastrophic structural failures. The precision of 96.8 ± 0.1% ensures minimal false alarms, reducing unnecessary interventions and maintaining production efficiency. Moreover, the 58.0 FPS inference speed enables real-time monitoring during welding processes, allowing for immediate quality assessment and corrective actions. The consistent high performance across varying IoU thresholds (mAP@0.5–0.95: 98.2 ± 0.1%) demonstrates the model’s exceptional stability and reliability under different detection standards and industrial conditions, making it highly suitable for deployment in diverse manufacturing environments.

### 6.2. Ablation Experiments

**Figure 9:**
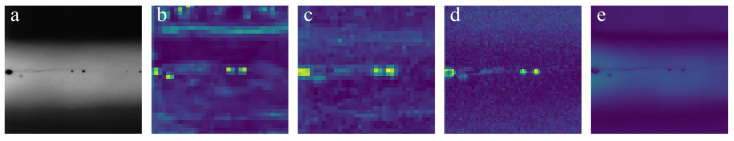
Model Learning Visualization. (**a**) Input. (**b**) HFPS level. (**c**) MPWC level. (**d**) Decoder and Head. (**e**) Output.

**Figure 10:**
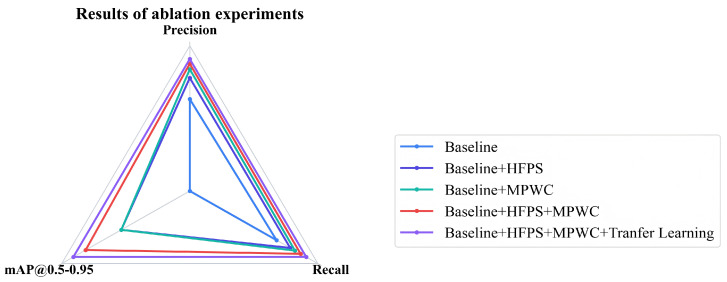
Model Performance Comparison Radar Chart.

To validate the effectiveness of the proposed WELD-DETR model, we conducted a systematic ablation study to quantitatively assess the contribution of each key component. As illustrated in Table 5, Figure 9 and Figure 10. The experimental results revealed that the baseline model achieved an mAP of only 89.7% and a precision of 91.7%, highlighting the limitations of traditional detection architectures in welding defect recognition. Qualitative analysis shows the baseline model consistently misses fine cracks and small pores due to its limited multi-scale representation capability. This outcome underscores the inherent challenges of conventional methods in handling the unique characteristics of welding images, including high noise levels, non-uniform lighting, and complex texture patterns. Particularly for welding defects with a wide range of sizes and variable shapes, traditional single-scale feature extraction mechanisms struggle to accurately detect both large slag inclusions and micron-sized cracks.

To provide deeper insight into the behavior of each component, we analyze characteristic errors of the ablated variants. The baseline model (row 1) consistently misses fine cracks and sub-pixel pores due to limited multi-scale representation capability. The HFPS-only variant (row 2) improves small defect detection but produces false positives in complex texture regions, indicating enhanced sensitivity at the cost of specificity. The MPWC-only variant (row 3) effectively identifies texture anomalies but generates incomplete boundaries for elongated defects and shows reduced performance on low-contrast defects, highlighting its strength in local feature extraction but weakness in global context integration. The model without transfer learning (row 4) shows significant performance degradation under noisy conditions, frequently misclassifying background textures as defects. These qualitative observations align with the quantitative results and clarify the specific limitations addressed by each component.

When the HFPS Structure was introduced, the model performance improved significantly, with mAP increasing from 89.7% to 94.1%, representing a 5.4% improvement. This enhancement underscores the crucial role of multi-scale feature fusion strategies. HFPS facilitates bidirectional feature propagation paths—both bottom-up and top-down—enabling information exchange and complementary fusion across different hierarchical feature maps. The lower-level feature maps retain the original high spatial resolution, crucial for precisely locating fine defects such as micropores, while higher-level maps, although lower in spatial resolution, contain rich semantic information and global contextual understanding. These high-level features effectively distinguish true defects from normal welding texture patterns, reducing false detection rates. However, the improved sensitivity comes at the cost of increased false positives in regions with complex weld textures, as observed in qualitative results. This hierarchical feature fusion mechanism allows the model to maintain sensitivity to fine details while enhancing its semantic discriminative power, particularly for boundary-blurred defects that are easily overlooked or misclassified under a single scale.

Upon independently integrating the MPWC module, the model’s mAP further increased to 94.7%, a 5.0% improvement. This enhancement is driven by MPWC’s parallel deployment of multiple wavelet convolution kernels at different scales, constructing a multi-resolution feature perception system. The multi-scale decomposition properties of wavelet transforms enable simultaneous analysis of welding images in both frequency and spatial domains. Large-scale kernels capture the overall morphological features and spatial distribution patterns of defects, medium-scale kernels focus on extracting gradient changes and contour information at defect edges, and small-scale kernels delve into fine texture details and microscopic structural features. This multi-kernel collaborative perception mechanism is particularly important for welding defect detection, as different defect types exhibit distinct scale characteristics. For example, cracks typically appear as elongated linear structures, requiring small-scale kernels to capture their extension and branching patterns, while slag inclusions manifest as larger blocky regions, necessitating large-scale kernels for understanding their overall distribution. Additionally, the directional selectivity of wavelet transforms makes MPWC more sensitive to the directional features of defects, which is critical for identifying cracks with specific directional extension.

However, qualitative analysis reveals that the MPWC-only variant exhibits certain limitations: it tends to produce incomplete defect boundaries, particularly for elongated cracks where global contextual understanding is crucial. The module’s strong focus on local texture features sometimes results in fragmented detections of continuous defects, and it shows reduced performance on low-contrast defects where intensity variations are subtle. These observations explain the quantitative gap between the MPWC-only configuration (94.7% mAP) and the full model (98.2% mAP), highlighting the importance of integrating MPWC with HFPS for comprehensive defect understanding.

When both HFPS and MPWC modules were combined, the model achieved a peak mAP of 97.3% and a precision of 96.2%. More importantly, the two modules exhibited a powerful synergistic effect—HFPS’s multi-scale feature fusion framework provided MPWC with richer and more comprehensive input feature representations, ensuring that each scale of convolution kernels operated on the most appropriate feature layers. In turn, the edge and texture features enhanced by MPWC were shared across different layers via HFPS’s feature propagation mechanism, forming a positive feedback loop of feature enhancement. This deep coupling enabled the model to build more robust and discriminative feature representations, especially for small defects that are prone to interference in complex backgrounds.

Finally, by incorporating a cross-condition training strategy based on transfer learning, the model’s performance peaked at 98.2% mAP, a 0.9% improvement over the configuration using only HFPS and MPWC. The success of this strategy lies in its phased training paradigm: initially pre-training on a large-scale general visual dataset to learn basic visual feature extraction capabilities; then conducting domain-adaptive training on an industrial-grade welding dataset, which encompasses five common defect types under various working conditions, such as different lighting intensities, welding materials, and surface treatment states; and finally applying an incremental learning strategy across conditions, enabling the model to gradually adapt to extreme conditions, such as high noise, strong reflections, and oil contamination. This is particularly important given the observed performance degradation of the non-transfer-learning variant under noisy conditions, where it frequently misclassifies background textures as defects. This progressive training method not only accelerates the model’s convergence speed and final performance but, more importantly, enhances its generalization ability and robustness in real-world industrial environments. The transfer learning strategy optimizes feature representation rather than increasing computational complexity, which is crucial for practical deployment, ensuring that the model meets industrial detection accuracy standards while maintaining real-time performance.

### 6.3. Comparison of Object Detection Results

**Figure 11:**
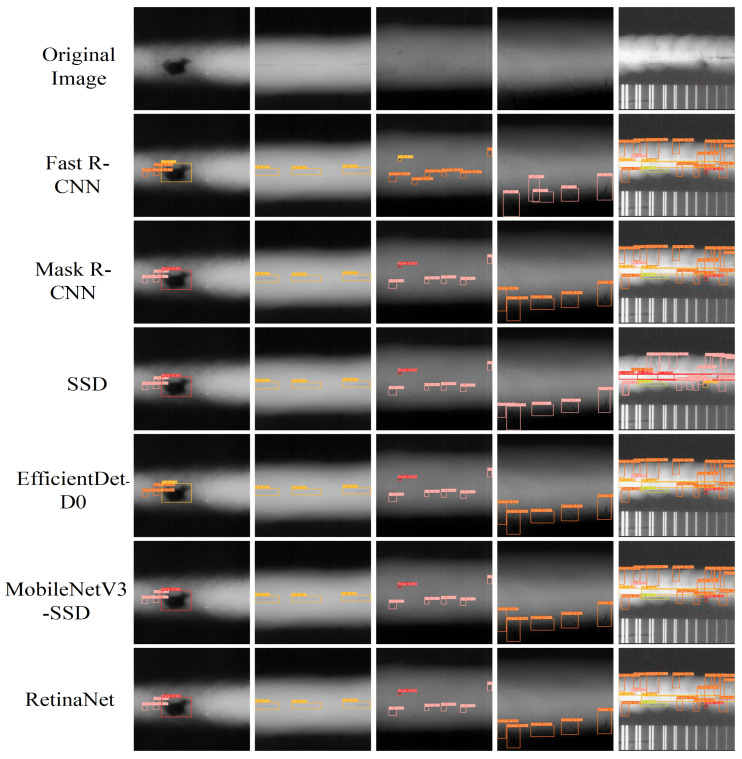
Comparison of Object Detection Results from Traditional Models. (Original Image, Fast R-CNN, Mask R-CNN, SSD, EfficientDet-D0, MobileNetV3-SSD, RetinaNet).

Figure 11 showcases the detection results from traditional object detection models such as Fast R-CNN, Mask R-CNN, SSD, EfficientDet-D0, MobileNetV3-SSD, and RetinaNet. These models, which primarily rely on region-based or multi-scale feature extraction techniques, generally exhibit a broader distribution of attention across the image. As a result, the bounding boxes they generate are often less tightly aligned with the detected objects, leading to reduced localization precision. For instance, Fast R-CNN and Mask R-CNN produce bounding boxes that are more generalized, especially in cluttered scenes or when objects overlap. While Mask R-CNN performs well in segmentation tasks by predicting pixel-wise masks, its bounding boxes often lack the fine-grained precision needed for accurate localization. EfficientDet-D0 and MobileNetV3-SSD are optimized for efficiency, but this efficiency comes at the cost of less detailed attention, meaning these models may struggle with precise object detection, particularly for small or closely spaced objects. RetinaNet, which uses focal loss to address class imbalance, provides better performance in detecting smaller objects but still cannot match the fine-grained attention seen in transformer-based models, such as WELD-DETR.

**Figure 12:**
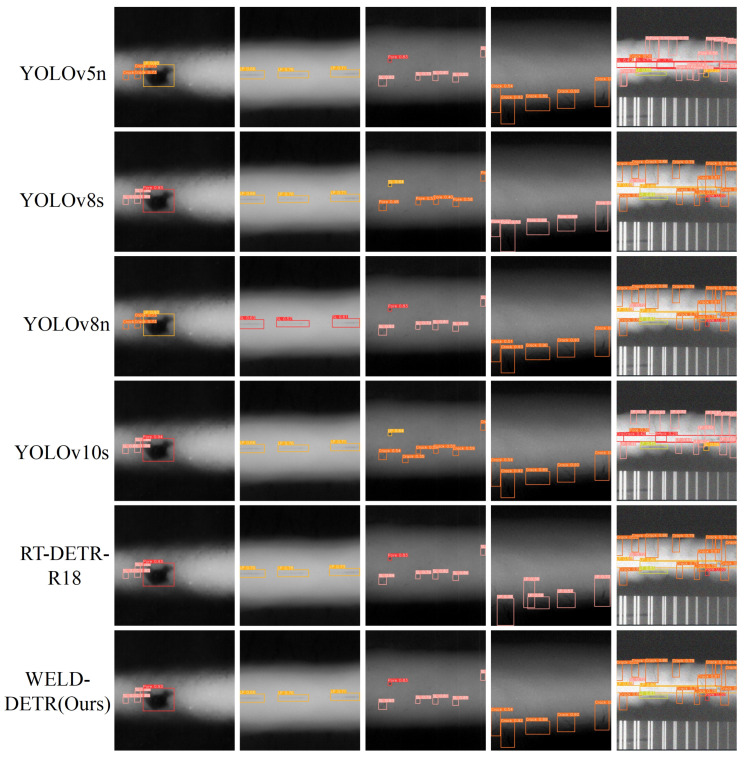
Comparison of Object Detection Results from YOLO and DETR Models. (YOLOv5n, YOLOv8s, YOLOv8n, YOLOv10s, RT-DETR-R18, WELD-DETR (Ours)).

Figure 12 then presents the performance of YOLO-based models (YOLOv5n, YOLOv8s, YOLOv8n, YOLOv10s) and RT-DETR-R18, alongside our WELD-DETR model. YOLO models, widely recognized for their speed, demonstrate broad attention distributions across the image. While this characteristic enables them to perform real-time detection efficiently, it also leads to less precise localization, particularly in challenging environments where objects are small, occluded, or have low contrast. The bounding boxes in YOLOv5n and YOLOv8s are broader, indicating that the model is allocating attention too generally. Although newer versions like YOLOv8n and YOLOv10s show improvements with slightly tighter bounding boxes, they still struggle with accurately localizing small or densely packed objects. In contrast, RT-DETR-R18, a transformer-based model, significantly improves upon YOLO by leveraging self-attention to capture global contextual information. This enables more precise bounding boxes that are better aligned with the contours of the detected objects. However, while RT-DETR-R18 shows improvements in localization, it still cannot match the level of accuracy achieved by WELD-DETR.

Our model, WELD-DETR, outperforms both YOLO and RT-DETR due to its advanced multi-scale feature fusion. WELD-DETR focuses more precisely on key regions, maintaining high localization accuracy even in complex, cluttered environments. The bounding boxes generated by WELD-DETR are more tightly aligned with the objects, indicating the model’s ability to concentrate attention on relevant regions and minimize unnecessary spread. This is especially noticeable in scenes with overlapping objects or weak contrasts, where WELD-DETR excels in handling occlusions and accurately detecting objects at various scales. The model’s transformer-based architecture, combined with its attention refinement techniques, makes it highly effective in scenarios requiring both speed and precision.

In conclusion, the comparison between WELD-DETR and other traditional or YOLO-based models highlights the significant advantages of our model in terms of both accuracy and precision. While traditional models and YOLO-based architectures excel in speed and efficiency, WELD-DETR offers a more robust solution for challenging detection tasks. Its ability to focus attention tightly on relevant regions and accurately localize objects, even in cluttered or complex environments, positions it as a superior model for real-world applications that demand high precision, robustness, and adaptability to varying object scales and scene complexities. This makes WELD-DETR a powerful tool for advanced object detection tasks, offering significant improvements over both conventional and contemporary detection models.

To complement the quantitative assessment, we performed extensive qualitative evaluation under diverse industrial scenarios. The HFPS structure demonstrates exceptional capability in handling multi-scale defects, maintaining fine-grained spatial information for micron-scale cracks while simultaneously capturing broader contextual semantics for voluminous slag inclusions. The MPWC module substantially improves discriminative capability in challenging imaging conditions through its multi-domain processing approach—the integrated wavelet transform effectively separates defect signatures from complex background patterns under low-contrast and high-noise situations. Particularly in environments with strong specular reflections or significant sensor noise, the complementary operation of HFPS and MPWC sustains reliable detection performance where conventional architectures exhibit substantial degradation (comprehensive visual evidence and systematic robustness analysis across varying illumination and noise levels are provided in Supplementary Material).

Notwithstanding these achievements, we recognize several inherent constraints. The framework’s generalization capacity may be compromised by domain shifts when encountering previously unseen welding materials exhibiting distinct radiographic characteristics. Furthermore, although our approach represents a substantial advancement in micro-defect detection, exceptionally faint defects exhibiting minimal contrast differentials continue to present detection challenges.

### 6.4. Object Detection Heatmaps

**Figure 13:**
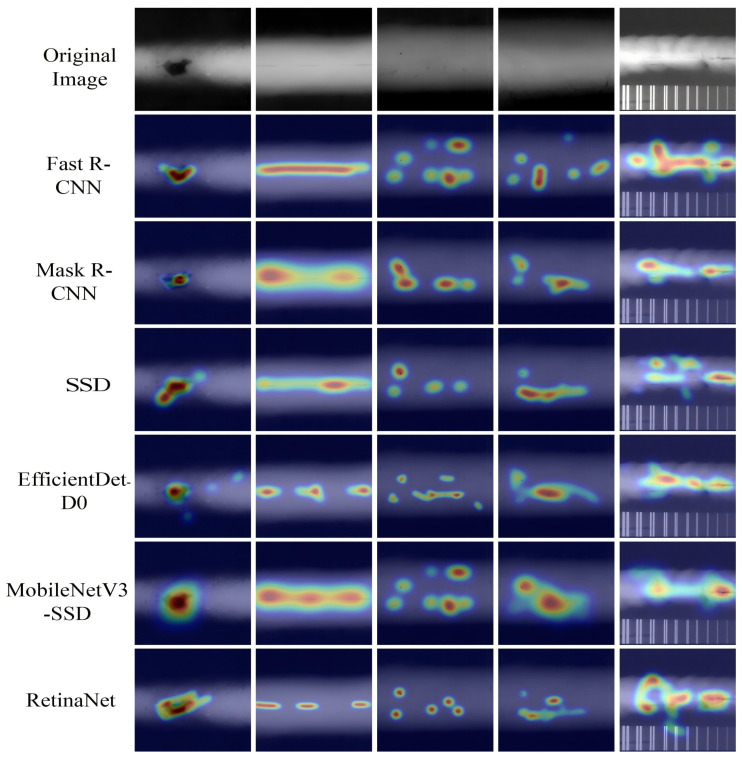
Object Detection Heatmaps for Traditional Models. (Original Image, Fast R-CNN, Mask R-CNN, SSD, EfficientDet-D0, MobileNetV3-SSD, RetinaNet).

**Figure 14:**
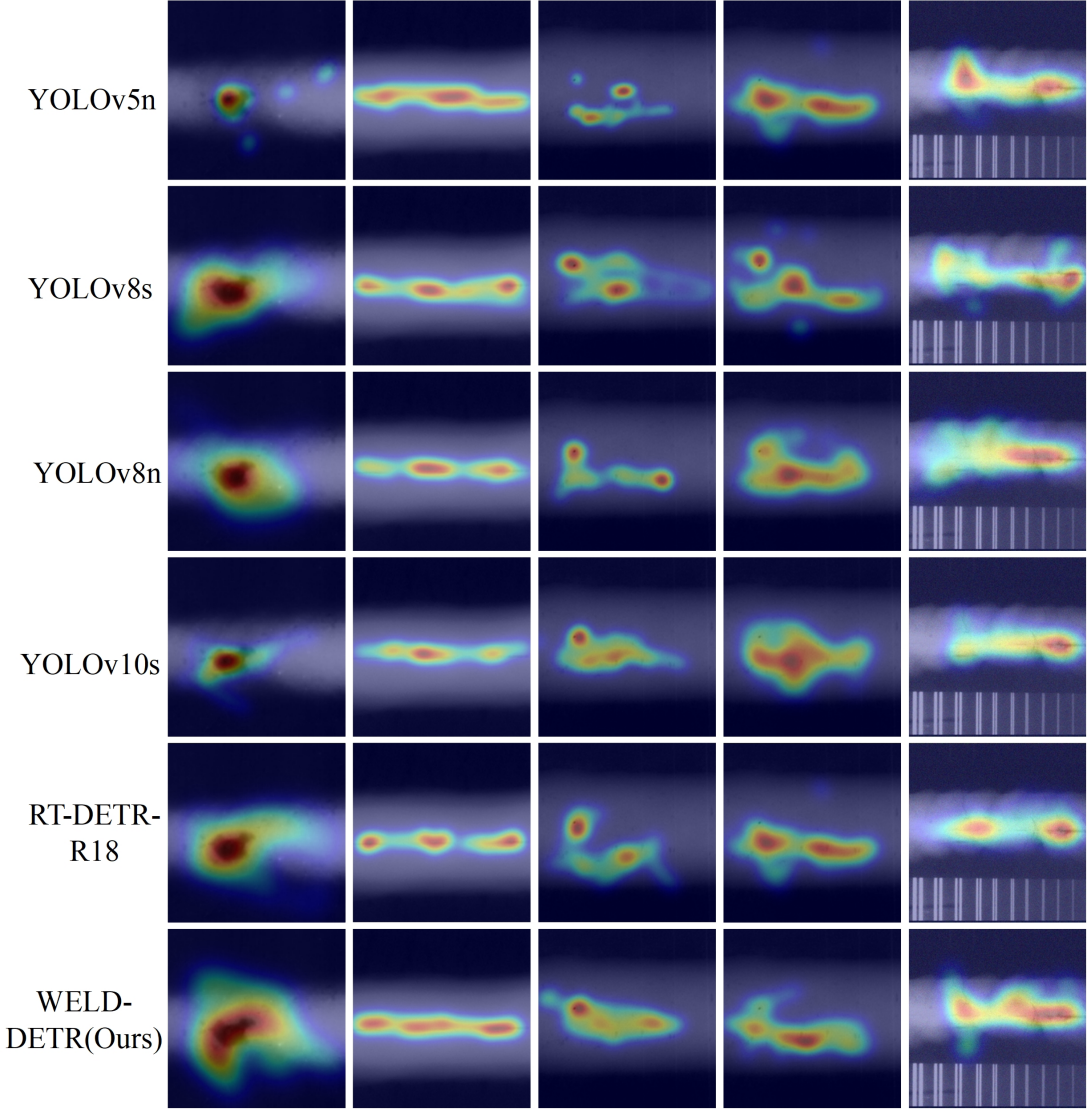
Object Detection Heatmaps for YOLO and DETR Models. (YOLOv5n, YOLOv8s, YOLOv8n, YOLOv10s, RT-DETR-R18, WELD-DETR (Ours)).

Figure 13 illustrates the heatmaps for traditional detection models, including Fast R-CNN, Mask R-CNN, SSD, EfficientDet-D0, MobileNetV3-SSD, and RetinaNet, while Figure 14 presents the heatmaps for WELD-DETR, along with other state-of-the-art models such as YOLOv5n, YOLOv8s, YOLOv8n, YOLOv10s, and RT-DETR-R18.

The heatmap visualizations reveal significant differences in how each model allocates attention to various regions of the image [40]. WELD-DETR, which incorporates an enhanced transformer-based architecture, stands out due to its superior ability to focus attention on both small and large objects with high precision. By integrating multi-scale feature fusion and a robust attention mechanism, WELD-DETR minimizes the spread of attention and enhances localization in challenging environments with complex backgrounds or occlusions. This refined attention is particularly evident in the heatmaps, where WELD-DETR demonstrates a concentrated focus on key regions of interest, making it more adept at detecting objects in cluttered or low-contrast settings compared to other models.

Traditional models like Fast R-CNN, Mask R-CNN, and EfficientDet-D0 exhibit more generalized attention patterns, often focusing less on the finer details of object boundaries, which can lead to decreased performance in detecting small or obscured objects. WELD-DETR addresses these challenges by leveraging a more sophisticated approach to feature aggregation and attention processing, allowing it to outperform these models in complex detection tasks, as reflected in the more focused and precise heatmap patterns.

In comparison, the YOLO-based models (such as YOLOv5n and YOLOv8n) show a broader distribution of attention, which, while efficient for real-time detection, may sacrifice localization precision, particularly in dense or overlapping scenes. RT-DETR-R18, another transformer-based model, also shows improved focus on objects, but the heatmaps indicate less fine-tuned attention compared to WELD-DETR, which benefits from our model’s specialized multi-scale feature extraction and attention refinement techniques.

The heatmaps highlight the distinct advantages of WELD-DETR, particularly in its ability to concentrate attention on relevant regions while minimizing unnecessary spread. This ability to refine attention leads to more accurate object detection in difficult environments, making WELD-DETR a powerful model for real-world applications where precision and robustness are critical. The comparative analysis underscores the effectiveness of WELD-DETR in overcoming the limitations of traditional and existing state-of-the-art models, particularly in challenging detection scenarios.

Our work demonstrates that the WELD-DETR framework, built upon the real-time RT-DETR baseline, achieves a superior balance of accuracy and speed for the specific demands of industrial weld inspection. While we focused on RT-DETR for its deployment-friendly profile, the core innovations of HFPS and MPWC—hierarchical feature fusion and multi-kernel wavelet-based perception—are modular by design. This opens a promising avenue for future research: integrating our feature-level modules into the encoder or backbone of other advanced DETR variants, such as Deformable DETR or DINO-DETR. Such a hybrid architecture could potentially combine the faster convergence and powerful decoding capabilities of these models with our method’s enhanced sensitivity to fine edges and textures. This would be particularly valuable for pushing the boundaries of defect detection in highly noisy, low-contrast environments where preserving high-frequency cues is paramount.

## 7. Conclusions

This paper presents WELD-DETR, a novel real-time welding defect detection framework that addresses critical challenges in industrial welding quality assurance through innovative multi-scale feature fusion and multi-kernel perceptual optimization techniques. Experimental results demonstrate that the proposed framework achieves state-of-the-art performance while maintaining real-time processing capability, showing strong potential for practical industrial deployment. The main conclusions are as follows:

(1) The HFPS effectively bridges the semantic gap between low-level, high-resolution features and high-level semantic representations. This hierarchical design allows the model to capture fine structural details without losing global context, significantly improving the detection of microscale defects such as cracks and pores that are often overlooked by conventional detection approaches. Experimental results indicate that HFPS contributes a 6.4% increase in mean Average Precision (mAP) for small-defect detection tasks, confirming its critical role in enhancing fine-grained recognition.
(2) The MPWC module enhances the network’s ability to extract multi-scale edge patterns and fine texture representations. This improves the model’s adaptability to defects with diverse morphological characteristics, such as irregular crack edges and varying pore sizes. Quantitative analysis shows that defect classification accuracy increases by 4.7% relative to standard convolutional backbones, highlighting the effectiveness of introducing wavelet-based multi-kernel perception into the architecture.
(3) Leveraging an industrial-grade dataset containing five common defect categories, the proposed transfer learning-based training strategy enhances robustness across diverse operating conditions. In cross-domain evaluations, the strategy reduces performance degradation under varying noise levels and illumination conditions, improving the model’s generalization capability. This ensures that WELD-DETR can adapt effectively to the complexities of real-world industrial environments.

Overall, WELD-DETR achieves an average mAP@0.5–0.95 of 98.2% while sustaining real-time inference at 58 FPS on an NVIDIA RTX 2060 GPU, striking a strong balance between accuracy and efficiency. The framework’s capability extends beyond laboratory settings, maintaining reliable performance under challenging industrial conditions including high noise and strong reflections.

It is important to acknowledge several limitations of the current study. First, the framework operates exclusively on 2D radiographic images and does not incorporate 3D volumetric data or time-series information that could provide additional defect characterization. Second, while the dataset covers five common defect types, its diversity is constrained by the specific welding materials and configurations used in our experimental setup. Third, the current implementation focuses on single-modality analysis and lacks integration with complementary non-destructive testing techniques such as ultrasonic testing or thermal imaging. These limitations, while not diminishing the current contributions, highlight opportunities for future enhancement.

While the current work demonstrates excellent performance on radiographic imagery, several directions merit further investigation. Future research will explore extending the approach to other material types and welding configurations through domain adaptation techniques. Integration with robotic inspection systems could enable closed-loop quality control, while multimodal sensing combining ultrasonic, thermal, and acoustic data may provide complementary defect information. Additionally, investigating uncertainty estimation and continual learning would enhance the framework’s long-term reliability in evolving industrial environments.

WELD-DETR represents a significant step forward in automated welding inspection. By effectively addressing key challenges in small defect detection, real-time performance, and industrial robustness, the framework provides both methodological innovations and practical solutions. The public release of datasets and source code will facilitate further advancement in intelligent non-destructive testing technologies.

## Figures and Tables

**Table 1:** Characteristics of Welding Defects in the Dataset.

Type	X-Ray Appearance	Proportion
Crack	Thin linear dark shadows with widths of 2–8 pixels, appearing as straight, curved, or branched configurations with high aspect ratios and sharp contrast boundaries	18.5%
Incomplete Penetration	Continuous dark bands positioned along the weld centerline, presenting as rectangular or strip-like formations with clear, well-defined boundaries and uniform gray values	22.3%
Pore	Well-defined circular or elliptical dark regions with smooth boundaries and uniform internal gray values, ranging from isolated single pores to clustered multiple arrangements	18.2%
Slag Inclusion	Irregularly shaped dark regions with fuzzy, indistinct boundaries and non-uniform internal gray values, exhibiting complex morphologies including blocky, strip-like, and dendritic patterns	25.2%
Incomplete Fusion	Two distinct parallel dark bands with strong geometric regularity, characterized by fixed spacing and linear alignment, typically appearing with consistent width and high contrast	15.8%

**Table 2:** Experimental Environment.

Parameter	Value
Operating System	Windows 10 (64-bit)
RAM	32 GB
GPU	NVIDIA GeForce RTX 2060
CPU	Intel(R) Core(TM) i7-10870H CPU @ 2.20 GHz
Python Version	3.8.5
PyTorch Version	1.10.0
CUDA Version	11.3

**Table 3:** Hyperparameter Settings.

Parameter	Value
Batch Size	16
Learning Rate	0.01
Optimizer	Stochastic Gradient Descent (SGD)
Momentum	0.937
Epochs	200

**Table 4:** Comparison of model performance.

Models	Precision P/%	Recall R/%	mAP@0.5–0.95/%	Speed (FPS)	Parameters/M
Faster R-CNN	88.5 ± 0.4	86.3 ± 0.5	82.0 ± 0.6	12.3	41.2
Mask R-CNN	87.9 ± 0.5	85.2 ± 0.7	80.5 ± 0.8	10.6	44.5
SSD	84.3 ± 0.6	81.6 ± 0.8	76.0 ± 0.9	45.1	28.3
EfficientDet-D0	89.2 ± 0.3	87.1 ± 0.4	83.5 ± 0.5	38.4	3.7
MobileNetV3-SSD	83.8 ± 0.7	80.5 ± 0.9	74.3 ± 1.0	60.2	5.8
RetinaNet	90.3 ± 0.3	90.5 ± 0.3	84.1 ± 0.4	18.6	36.4
YOLOv5n	88.6 ± 0.4	86.5 ± 0.5	81.9 ± 0.6	55.3	3.8
YOLOv8s	92.4 ± 0.2	90.7 ± 0.3	90.8 ± 0.3	86.7	11.2
YOLOv8n	93.0 ± 0.2	92.4 ± 0.2	85.5 ± 0.4	94.2	3.2
YOLOv10s	93.1 ± 0.2	91.4 ± 0.3	94.5 ± 0.2	93.2	8.3
RT-DETR-R18	93.5 ± 0.2	93.8 ± 0.2	92.6 ± 0.2	72.4	19.5
**WELD-DETR (Ours)**	**96.8 ± 0.1**	**94.5 ± 0.2**	**98.2 ± 0.1**	**58.0**	**15.6**

All detection metrics (Precision, Recall, mAP) are reported as mean ± standard deviation over 5 independent runs with different random seeds. Speed and Parameters are fixed values.

**Table 5:** Results of ablation experiments.

Baseline	HFPS	MPWC	Transfer Learning	Precision P/%	Recall R/%	mAP@0.5–0.95/%
✓	×	×	×	91.7	90.8	89.7
✓	✓	×	×	94.4	92.5	94.1
✓	×	✓	×	95.5	93.1	94.7
✓	✓	✓	×	96.2	93.8	97.3
✓	✓	✓	✓	**96.8**	**94.5**	**98.2**

A checkmark (✓) indicates the presence of the component or a positive outcome, while a cross (×) indicates its absence or a negative outcome.

## Data Availability

All supplementary data are available in the Supplementary Materials section.

## Supplementary Materials

The following supporting information can be downloaded at: https://github.com/yubojian123/WELD-DERT (accessed on 10 October 2025).
